# Physical Activity for the Treatment of Chronic Low Back Pain in Elderly Patients: A Systematic Review

**DOI:** 10.3390/jcm9041023

**Published:** 2020-04-05

**Authors:** Gianluca Vadalà, Fabrizio Russo, Sergio De Salvatore, Gabriele Cortina, Erika Albo, Rocco Papalia, Vincenzo Denaro

**Affiliations:** Department of Orthopaedic and Trauma Surgery, University Campus Bio-Medico of Rome, 00128 Rome, Italy; g.vadala@unicampus.it (G.V.); s.desalvatore@unicampus.it (S.D.S.); g.cortina@unicampus.it (G.C.); e.albo@unicampus.it (E.A.); r.papalia@unicampus.it (R.P.); denaro@unicampus.it (V.D.)

**Keywords:** chronic low back pain, elderly, old aged patients, physical therapy, physical activity, walking, global postural rehabilitation, cycling, hydrotherapy, yoga

## Abstract

Chronic low back pain (CLBP) affects nearly 20–25% of the population older than 65 years, and it is currently the main cause of disability both in the developed and developing countries. It is crucial to reach an optimal management of this condition in older patients to improve their quality of life. This review evaluates the effectiveness of physical activity (PA) to improve disability and pain in older people with non-specific CLBP. The Preferred Reporting Items for Systematic reviews and Meta-Analyses (PRISMA) guidelines were used to improve the reporting of the review. Individual risk of bias of single studies was assessed using Rob 2 tool and ROBINS-I tool. The quality of evidence assessment was performed using GRADE analysis only in articles that presents full data. The articles were searched in different web portals (Medline, Scopus, CINAHL, EMBASE, and CENTRAL). All the articles reported respect the following inclusion criteria: patients > 65 years old who underwent physical activities for the treatment of CLBP. A total of 12 studies were included: 7 randomized controlled trials (RCT), 3 non-randomized controlled trials (NRCT), 1 pre and post intervention study (PPIS), and 1 case series (CS). The studies showed high heterogeneity in terms of study design, interventions, and outcome variables. In general, post-treatment data showed a trend in the improvement for disability and pain. However, considering the low quality of evidence of the studies, the high risk of bias, the languages limitations, the lack of significant results of some studies, and the lack of literature on this argument, further studies are necessary to improve the evidences on the topic.

## 1. Introduction

Low back pain (LBP) is a common symptom that can improve spontaneously within a few weeks. However, about 2–7% [1] of cases may evolve into chronic low back pain (CLBP) that may lead to significant disability. Age is a well-known risk factor for CLBP in association to [2,3], psychological distress, inactivity, social environment, comorbidity, gender, genetic, and prior work exposure. CLBP affects approximately 20–25% of the elderly population (older than 65 years) [4], and it currently is the main cause of disability both in the developing and developed countries [5,6]. It increases linearly from the third decade of life affecting more women than men [7]. After a single episode of LBP, there is a higher risk to become recurrent [8]. CLBP, that is one of the most important conditions that leads to work-related disability, has dramatic consequences on the costs for the health system [9]. It is defined by the location of pain between the lower rib margins and the buttock that lasts for more than 12 weeks [10,11] and it can be often accompanied by neurological symptoms in the lower limbs (i.e., sciatica). Causes of CLBP can be distinguished into specific (degenerative process to the spinal segments of the lumbar spine such as lumbar spinal stenosis, spondylolisthesis, or disc herniation) [12] or non-specific, apparently when there is no underlying source of pain [13]. Among patients affected by LBP in primary care, patients affected by CLBP represent the greatest part (over 85%) [14]. CLBP in older adults has multifactorial causes, including both biological (insufficient muscle function around the spine [15]), and psychosocial factors [16] and, especially in the older adults it can lead to a severe reduction of independence and performance of normal daily activities [17].

Thus, it is crucial to reach an optimal management of this condition in older patients in order to improve their quality of life. However, limited evidence is available about the effectiveness of commonly recommended treatments for the older patient with CLBP. Paeck et al. showed that only a few clinical trials published in the literature were focused on older people. In fact, most studies include people younger than 65 years [18]. However, not all treatment options normally indicated for young people can also be pursued in the elderly population, since there may be other comorbidities, such as osteoporosis, that can limit their applicability.

Clinical practice guidelines for CLBP recommend physical activity (PA) as one of the most used interventions based on its biological rationale [19] and since it is easily applicable and low cost [20,21,22]. PA improves functions, mobility, quality of life, and some psychological distress that can be often found in older adults. Moreover, PA can improve social and work participation, coping strategies, and reduces fear-related beliefs regarding CLBP [23]. In the same way, physical inactivity is significantly correlated with the worsening of several chronic conditions including type 2 diabetes mellitus, congestive heart failure, and cognitive disorders such as depression and neurodegenerative diseases [24]. Therefore, PA can be useful and have positive effects on older patients with CLBP and other chronic conditions [25].

In the current review, PA is defined as a supervised activity program including general physical fitness programs, total body cardiovascular exercises, back schools, and specific techniques aimed at increasing single muscle strength or stretching such as Pilates, McKenzie, Feldenkrais, Tai Chi, or aquatic physiotherapy/hydrotherapy. The aim of this systematic review is to evaluate the effectiveness of PA in improving disability and pain in elderly patients with non-specific CLBP, comparing the results with groups of patients treated through manual therapy and other therapies that include non-physical intervention (advice to keep active) and untreated groups.

## 2. Materials and Methods

We focused our research on studies concerning PA as a treatment for CLBP in elderly patients. The Preferred Reporting Items for Systematic reviews and Meta-Analyses (PRISMA) guidelines were used to improve the reporting of the review. The Grading of Recommendations Assessment Development and Evaluation (GRADE) [26] approach was used to assess the quality of evidence of the articles that include full data.

### 2.1. Eligibility Criteria

#### 2.1.1. Study Inclusion Criteria

- Peer-reviewed studies of each level of evidence according to Oxford Classification. We included randomized clinical trials (RCT) and non-randomized controlled studies (NRCT) designs such as observational studies (OS), pre-post interventional studies (PPIS), and case-series studies (CS). We excluded case reports, technical notes, letters to editors, instructional courses, in vitro studies, cadaver investigation, systematic reviews, and meta-analyses.
- Studies including elderly patients (mean age > 65 years) suffering by CLBP (at least > 3 months).
- Clinical outcomes (disability and pain) of patients treated with PA (cardiovascular or aerobic) or exercise programs that included loaded (against gravity or resistance) as a component. To define a study as eligible, it had to include at least one pain assessment or one disability assessment. The disability outcome needed to be evaluated by one or more of the following scales: 36-Item Short Form Health Survey (SF-36) Version 1.0 and 2.0 (SF-36); Roland Morris Disability Questionnaire (RMDQ); Oswestry Disability Index (ODI); and Back function (FFBH-R) [27]. The pain outcome had to be evaluated by one or more of the following scales: Numerical pain rating scale (NRS); Global Rating Change (GRC); Patient Pain Questionnaire (PPQ); and Visual rating scale (VRS).
- Only articles written in English and Italian languages were included.

#### 2.1.2. Study Exclusion Criteria

- Studies with a mean age of patients < 65 years old;
- Studies in which PA was a part of a multidisciplinary program;
- Studies including participants who had physical problems that did not allow them to perform PA (diabetes untreated, muscle-skeletal problems, postural problems, neurological diseases, cardiovascular conditions).

### 2.2. Search Protocol 

The following articles were screened from inception to March 2019: Medline, Scopus, CINAHL, EMBASE, and CENTRAL. For the search strategy we decided to use the following keywords: “low back pain” OR “chronic low back pain” AND “physical activity” OR “physical therapy” AND “elderly” OR “old aged” OR “older age” AND “Meziere” AND “Souchard” AND “global postural rehabilitation” “Feldenkrais” AND “McKenzie” AND “back school program” AND “Tai-Chi” AND “Pilates” AND “water therapy” OR “hydrotherapy” OR “balneotherapy” OR “hydrokinesis.” We used the keywords isolated or combined. We searched for more studies among the reference lists of the selected papers and systematic reviews.

### 2.3. Study Selection 

**Figure 1:**
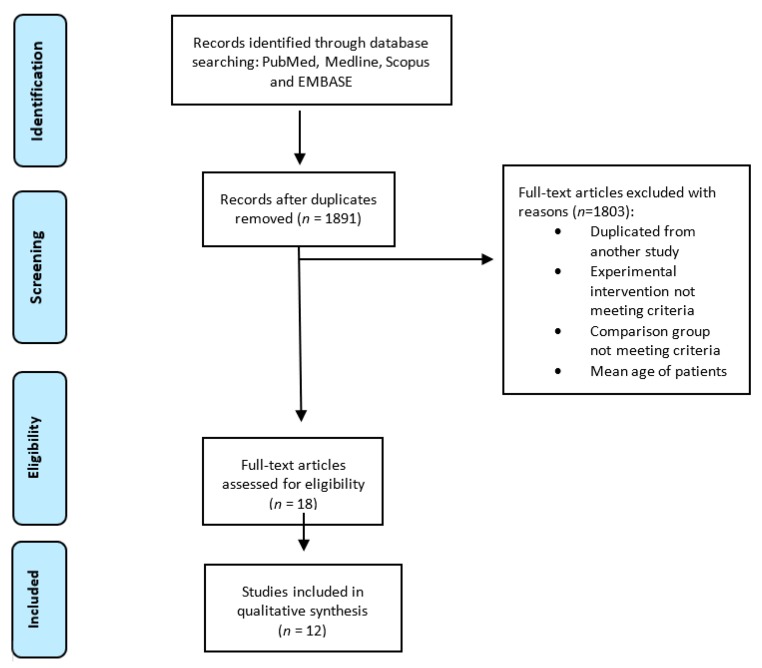
Preferred Reporting Items for Systematic reviews and Meta-Analyses (PRISMA) flow diagram.

We accepted only English and Italian publications. The initial search of the article was conducted by two reviewers (D.S.S. and C.G). They used the protocol of search previously described to identify literature. In case of disagreements, the consensus of a third reviewer (R.F.) was asked. The researchers used the following research order. Titles were screened first, then abstracts and full papers. A paper was considered potentially relevant and its full text reviewed if, following discussion between the two independent reviewers, it could not be unequivocally excluded on the basis of its title and abstract. The full text of all papers not excluded on the basis of abstract or title was evaluated. The number of articles excluded or included were registered and reported in a PRISMA flowchart (Figure 1). For designing the PRISMA we followed the rules by Moher et al. [28].

### 2.4. Data Extraction

Data were extracted on: author, n of participants, year of study, content of intervention and control group, follow-up, outcomes (disability and pain), and mean age.

### 2.5. Quality of Evidence

To estimate the potential bias that were most relevant for the study, we used the following tools: the Cochrane tool for assessing risk of bias in randomized trials (RoB 2 tool) [29] (Table 1) and the Risk Of Bias In Non-randomized Studies of Interventions (ROBINS-I) [30] (Table 2). In order to avoid imprecisions, the elected papers were rated independently by two reviewers (E.A. and S.D.S.) and verified by a third (G.V.). We used the GRADE approach (Table 3 and Table 4) to rate the overall quality of evidence. However, only six articles [31,32,33,34,35,36] showed full post-treatment data, therefore it was not possible to assess all the studies included using GRADE approach. The GRADE approach classifies the quality of evidence for each outcome grading the following domains: study design, risk of bias, inconsistency, indirectness, imprecision, publication bias, magnitude of the effect (not assessed in this study), dose-response gradient (not assessed in this study), and influence of all plausible residual confounding (not assessed in this study). The quality of evidence was then classified as follow:

- High Quality of Evidence: among 75% of articles included are considered with low risk bias. Further researches are useful to change either the estimate or confidence in results.
- Moderate Quality of Evidence: one of the GRADE domains is not met. Further studies are required to improve the quality of the study and the evidence.
- Low Quality of Evidence: two of the GRADE domains are not met. Further research is very important.
- Very Low Quality of Evidence: three of the GRADE domains are not met. The results of the study are very uncertain. In the case of studies with a sample size inferior to 300 subjects the quality of the study is considered very low if there was also a high risk of bias (assessed with different tools. In our study we used Rob2 and ROBINS-I).

The outcomes assessed were improvement in pain and disability, both evaluated at the end of the treatment. Follow-up were different and ranged from 1 month to 48 months. Furthermore, the outcomes were subgrouped into RCTs, NRCTs, and other studies (pre-post intervention and case series).

## 3. Results

### 3.1. Study Selection

We created a flow-chart diagram according to the PRISMA protocol that shows the selection process of the studies (Figure 1). We found a total of 2173 studies (no additional studies were found in gray literature). We obtained 1891 studies when the duplicates were removed. Of the 1891 studies, 1709 articles were excluded from our study through the title screening. We assessed the abstracts of 182 articles and we excluded 94. Then, 88 full-text articles were screened. Out of these studies, 76 were excluded for the following reasons: mean age of patients < 65 years old (*n =* 64); experimental intervention not meeting the inclusion criteria *(n =* 8), and comparison group not meeting the inclusion criteria (*n* = 4). After this process, we included 12 articles in our study. No unpublished studies were retrieved.

### 3.2. Study Characteristics

A description of the characteristics of the studies that was considered eligible for this review is reported in Table 5. A total of 12 articles were selected for this systematic review. We included 7 RCT of I level of evidence (LOE), 3 NRCT (3 OS of II LOE), 1 PPS of III LOE, and 1 CS of IV LOE. Studies were published between 1992 [37] and 2016 [31].

Based on the data of the included studies, a total of 1581 patients were treated for CLBP. The mean age of patients at the time of treatment was 71.88 ± 3.01 and ranged between 67.5 [36] and 76.0 [42].

The outcome measures used in these studies included: (3 studies) 36-Item Short Form Health Survey (SF-36) Version 2.0 (SF-36); (3 studies) Roland Morris Disability Questionnaire (RMDQ); (3 studies) Oswestry Disability Index (ODI); (2 studies) Numerical pain rating scale (NRS); (1 study) SF-36 Version 1.0; (1 study) Patient Pain Questionnaire (PPQ); (1 study) Global Rating Change (GCR); (1 study) Visual rating scale (VRS); and (1 study) Back function (FFBH-R) [27].

The studies cited in this review show high heterogeneity in terms of study design, interventions, and outcome variables. The results are presented descriptively, focusing on disability and pain and further issues of potential interest. In general, post-treatment data showed a moderate range of improvement for disability and pain. Otherwise, these results need to be evaluated carefully due to the high risk of bias and the high heterogeneity of the studies included.

### 3.3. Methodological Quality

The Rob2 tool for RCT and ROBINS-I tool for NRCT, pre-post intervention and case-series were used to assess the methodological quality of each study. For RCT we found three studies with an overall risk identified as “some concerns,” 3 as “high risk,” and 1 as “low risk”. Concerning the NRCT we found 1 study with an overall risk of bias identified as “critical” [38] and 2 studies as “moderate” [34,37]. We assessed the pre-post intervention study with an overall risk of bias identified as “serious” [41]; instead the case series was identified as “moderate” [42].

The quality of evidence of the studies included in GRADE ranges from low to moderate. All the studies, except one [34], have a small sample (n < 300). Methodological quality assessments of each study are summarized in Table 1 and Table 2. The quality of evidence of full data trials was performed using GRADE approach (Table 3 and Table 4). The analysis of the data of the study was reported using the mean difference between studies. RevMan5 (version 5.3) was used to calculate the mean difference of the included studies. Because of the lack of post treatment results in some studies, we decided to perform a systematic review and not a meta-analysis. We report the outcomes of each study in Table 5.

### 3.4. Results of Individual Studies

The intervention methods are usually well described in all the included studies. High heterogeneity in the type of PA was reported in all the studies. We included all types of PA (walking [32,35], back school and hydrotherapy [39], isotonic resistance exercises [40] yoga and qijong [31], TOTXR [33] and LEXTR). The authors divided the description of intervention per outcome (pain and disability) in three subgroups (randomized controlled trials, non-randomized controlled trials, and other studies, including pre-post intervention and case series).

#### 3.4.1. Randomized Controlled Trials (RCTs)

Seven RCTs were included. They were divided per outcome: 2 studies [36,40] examined the improvement in pain (measured by NRS and VRS); 5 studies [31,32,33,35,39] assessed the disability outcome (measured by ODI, RMDQ, PPQ, FRI, FFBH-R, and SF-36). Single studies were assessed for risk of bias using Rob2 tool. Two studies were classified as “high risk,” three as “some concerns,” and one as “low risk.” It was possible to include only 5 articles in GRADE analysis [31,32,33,35,36]. The overall quality of evidence in these studies ranges from “low” to “moderate” according to GRADE. The quantitative effect estimate was reported as mean difference between and within studies (when possible). This heterogeneity among studies and the low quality of evidence could lead to an overestimation of the results. The results of the outcome of the other studies are reported in Table 5.

##### Outcome: Pain

Two RCTs studies [36,40] presented data on pain at the end of the treatment. The authors used NRS and VRS to evaluate the improvements in pain. Follow-up was 3 months in the study carried out by Holmes et al. [42] and 4 months in the study by Vincent et al. [36]. At the end of the treatment, they both reported a reduction of pain in the group treated by PA (isotonic resistance exercises in Holmes et al. [42] group and TOTXR and LEXTR in Vincent [36] group). The study by Holmes et al. was classified as “high risk,” and the risk of bias of the study by Vincent et al. was assessed as “some concern” using Rob2 tool. The study by Vincent et al. [36] was assessed as “moderate” quality using GRADE analysis. It was not possible to evaluate the overall quality of the other study according to GRADE [26] because of the lack of data. Otherwise, in both articles it was reported an improvement in pain evaluated by NRS and VRS. Vincent et al. [36] reported a better NRS in the intervention group compared to the control group at the end of the treatment (MD −1.73, 95% C.I. −3.11 to −0.35, *p* = 0.01). Holmes et al. [42] reported a difference from 5.3 to 2.1 points in VRS from the beginning to the end of the treatment (no full data were reported concerning to control group results). Otherwise, the authors reported an improvement in pain between the intervention and the control group, but this was not statistically significant (*p* > 0.05). The results of the outcome of the other studies are reported in Table 5.

##### Outcome: Disability

Five RCT studies [31,32,33,35,39] presented data on disability at the end of the treatment. The authors used ODI, RMDQ, SF-36, PPQ, FRI, and FFBH-R to assess the improvements in disability. Follow-up was heterogenous: 1 month for Tsatsakos et al. [35]; 1.5 months for Ferrel et al. [32]; 3 months for Teut et al. [31] and Costantino et al. [41]; and 4 months for Vincent et al. [33]. At the end of the treatment, all studies reported an overall improvement in disability. The PA program was different between studies (walking [32,35], back school and hydrotherapy [39], yoga and Qijong [31] and TOTXR [33]). In the study by Ferrel et al. [32] the control group was constituted by the hydrotherapy group and not by a no-intervention group as in the other studies. Also, in this study they reported an overall increase in disability in both groups. The studies by Tsatsakos et al. and Ferrel et al. were classified as “high risk,” Teut et al. as “low risk,” Costantino et al. and Vincent et al. as “some concern” using Rob2 tool. It was not possible to assess the quality of evidence of the study by Costantino et al. [41] because of the absence of a “no-intervention” control group. The overall quality of the other 4 studies [31,32,33,35] was evaluated as “low” according to GRADE [26]. In specific, the authors divided the studies into two subgroups: RCTs measured by ODI and RCTs measured by SF-36. We used only these scales since they were reported in all studies. We found a reduction of disability evaluated by ODI (MD −1.24, 95% C.I. −1.94 to −0.54; *p* = 0.0005 *). Moreover, an improvement of SF-36 in patients treated by PA was reported (MD 2.88, 95% C.I. −3.30 to 9.06, *p* = 0.36). Costantino et al. [41] observed a highly significant statistical difference of SF-36 (13.30 ± 1.44, *p* < 0.001 *), measured in both intervention groups (back school and hydrotherapy) at the end of the treatment. The results of outcome of the other studies are reported in Table 5.

#### 3.4.2. Non-Randomized Controlled Trials (NRCT)

We included in our review three NRCT [34,37,38] studies. They were divided per outcome: 2 studies [34,37] examined the improvement in pain (measured by GRS and PPQ); 1 study [38] assessed the disability outcome (measured by RMDQ). The latter study did not have a control group. Single studies were assessed for risk of bias using ROBINS-I tool [30]. Two studies [34,37] were classified as “moderate” overall risk and one [38] as “critical.” Because of the lack of data, it was possible to assess the quality of evidence, according to GRADE, only of the study by Hicks et al. [34] classifying as “low.” The quantitative effect estimate of this study was reported as mean difference between groups. The high heterogeneity among studies and the low quality of evidence could lead to an overestimation of the results. The results of outcome of the other studies were reported in Table 5.

##### Outcome: Pain

Two NRCT studies [34,37] presented data on pain at the end of the treatment. The authors used GRS and PPS to evaluate improvements in pain. Follow-up was 1 month in the study by Khalil et al. [37] and 12 months in the study by Hicks et al. [34]. At the end of the treatment they both reported a reduction of pain in the group treated by PA (strengthening and stretching programs [34] and isotonic and isokinetic progressive resistive exercise [37]). The overall quality of the study by Hicks et al. [34] was evaluated as “low” according to GRADE [26]. The study by Khalil et al. [37] was classified as “moderate” risk of bias using ROBINS-I tool. In specific, Hicks et al. [34] reported an improvement in pain after the treatment in the intervention group compared to controls measured by GRC (MD-1.00, 95% C.I. −1.53 to −0.47, *p* = 0.006 *). Khalil et al. [37] also reported a reduction of pain measured by pain scale (1–10) from 5.5 to 3.3 (*p* < 0.01 *), but no data concerning to control group were found.

##### Outcome: Disability

One NRCT study [38] presented data on disability at the end of the treatment. The authors used RMDQ to evaluate improvements in disability. Follow-up was 2 months. One important limitation in the study was the lack of a control group. However, at the end of the treatment the authors concluded by reporting an improvement in disability in the group treated by PA (stretching and resistance exercises [38]). The risk of bias of this study was evaluated using ROBINS-I and it was classified as “serious.” Beissner et al. [39] reported a reduction of disability measured by RMDQ scale in patients treated by PA (−5.29 points, *p* < 0.001 *).

#### 3.4.3. Other Studies (Pre-Post Intervention and Case Series)

One pre-post intervention study [41] and one case series [42] presented data on disability at the end of the treatment. The authors used respectively SF-36 and ODI to evaluate improvements in disability. One important limitation of these studies was the lack of a control group. Because of the lack of a control group it was not possible to classify the evidence of these studies according to GRADE. Otherwise, the study by Mailloux et al. [38] was classified as “moderate” risk and Iversen et al. [40] as “serious” according to the ROBINS-I tool.

##### Outcome: Disability

Two studies [41,42] presented data on disability at the end of the treatment. They reported an improvement in disability in the groups treated by PA (cycling [41], stretching, resistance training, and endurance activities [42]). Follow-up was respectively 48 months in the study by Mailloux et al. [38] and 3 months in the study by Iversen et al. [40]. Iversen et al. [40] reported a non-statistically significant improvement in physical function measured by SF-36 of 7.2 points (*p* = 0.6). Mailloux et al. [38] reported a reduction of ODI from 28% ± 17 to 16% ± 13 (*p* = 0.001 *) in the intervention group after 48 months of follow-up. On the other hand, it was reported only a reduction of ODI from 38 ± 17 to 25 ± 17 (*p* = 0.001 *) in the control group.

## 4. Discussion

CLBP currently affects approximately one-fifth of the global population [43,44]. Cayea et al. reported that 36% of older adults aged 65 years or more are affected by at least one episode of this condition per year, of which 21% reported moderate or intense pain representing an important priority for the health system. In the literature, as confirmed by Paeck et al. [16], there is a lack of studies on CLBP in the elderly. In fact, most of the studies on CLBP treatment options are focused on the so-called “working age” and this calls into question the reliability of several treatment options in the older population, especially because in the older age we can often find several comorbidities that may limit the rehabilitation.

In our systematic review, we screened the recent literature (1992–2018) with the aim to assess the effectiveness of PA to improve disability and pain in the elderly population affected by non-specific CLBP comparing it to no treatment and other conservative treatments. Indeed, 12 studies were included at the end of the search process. Among these, only 5 RCTs with an overall quality of evidence that ranges from “low” to “moderate” and 1 NRCT of “low” quality could be assessed according to GRADE approach. The quality of the other studies was evaluated by Rob2 for RCT and ROBINS-I for the other study types. The lack of data in some articles, and the poor literature among this topic could lead to low quality of evidence. Our research highlighted that older patients with CLBP treated with PA showed an overall pain and disability improvement in the majority of the studies. Otherwise, these conclusions need to be taken carefully, considering the high risk of bias, the low quality of evidence of the literature, and the languages limitations of this study (only English and Italian articles were included). Because of these limitations and the absence of high-quality literature, we decided to perform only a systematic review of the literature and not a meta-analysis.

However, the extreme variability of type, duration, intensity, and execution modality of the proposed PA, the different body district on which PA were focused on in each different program and the compliance of the patients, are important variables that make it impossible to recommend a specific protocol in the elderly population. This lack of standardization was also confirmed by Airaksinen et al. [18] that found a considerable variety of PA, such as stretching, aerobic exercises, or muscle reconditioning.

In this systematic review, we analyzed different PA protocols, based on walking [35], cycling [41], back school exercises [39], hydrotherapy [39], Yoga and Quigong [31], endurance, resistance, stretching and strengthening exercises [33,37]. Regarding the trained muscle groups, we found that most of the included studies were focused on abdominus muscles [40], iliopsoas, hamstring, gastrocnemius, quadriceps, hip flexors, abductor/adductor muscles of the hip and erector spinae muscles [37].

Regarding the 4 studies evaluating pain (2 RCTs and 2 NRCTs), they showed that both lumbar isotonic resistance exercise cycles and abdominal, thoracolumbar and upper limb isotonic and isokinetic strengthening exercises, improve pain in elderly patients with CLBP. In their RCT, Vincent et al. [32] also reported, at a 4-months follow-up, an improvement in walking speed and endurance. This finding confirms that the physical treatment of CLBP might be focused not only on the lumbar muscles but also on the lower limbs and thorax (exercises for breathing muscle districts [39]). Otherwise, one study [40] reported an improvement in pain, but not statistically significant if compared with the control group (*p* > 0.05).

The studies which assessed disability (5 RCTs, 1 NRCT, 1 pre-post intervention and 1 case series) confirmed that walking, back school exercise, hydrotherapy, yoga and Qijong, bicycle program, strengthening and stretching program, and combined PA and cognitive-behavioral program improve the functional performances of elderly people with CLBP. However, because of the high heterogeneity of the studies, we found a significant reduction of disability evaluated by ODI (*p* = 0.000 5*), but the improvement of SF-36 in patients treated by PA was not significant (*p* = 0.36). Moreover, we also found an improvement in patients treated by different types of PA such as back school and hydrotherapy [39] (*p* < 0.001 *) at the end of the treatment.

Other important concerns are compliance and motivation of the patient that may represent decisive parameters during CLBP treatment in the elderly. Beissner et al. [39] emphasized an interesting treatment option represented by the cognitive-behavioral therapy (CBT) in association with PA to reduce symptoms in patients with CLBP. This novel treatment is becoming increasingly important. In a recent systematic review, Vitoula et al. [45] highlighted that CBT was effective in patients with CLBP, especially in reducing pain perception and helping them to improve their functionality. Furthermore, the review showed that better outcomes can be achieved when treatments are personalized. This represents a remarkable issue. In fact, several studies included in our research [34,38,39] showed that patients that maintain a prolonged compliance to the rehabilitation protocols and were highly motivated had better outcomes in pain relief and function outcomes.

It is crucial to focus on the biological effects of PA [46,47]. One major limit to perform PA in old-aged patients is the sarcopenia, defined as a loss of muscle mass (lean body mass) with a reduction of muscle function [48]. This process represents a specific condition of normal energy balance in the elderly, with an increase in body fat percentage. Limb surgery postoperative period, disuse, endocrine diseases (such as diabetes type II), and uncontrolled nutrients intake lead to sarcopenia [49]. This condition could lead to a frailty status, with a reduction of PA [50]. Landi et al. [51] conducted a review of the literature reporting that PA has an important role in the reduction of sarcopenia in old-aged people. PA could also increase irisin [52] and osteocalcin [53]. The former is a hormone-like myokine produced by skeletal muscle during PA [54]. Irisin can induce thermogenesis from brown adipocytes. This protein has also an effect in the control of bone mass, with positive effects on cortical mineral density. It is also demonstrated that irisin plays a crucial role in the reduction of sarcopenia in old people [55,56]. Osteocalcin is a bone-derived hormone-like protein. It could favor physiological functions increasing the bone formation [57], regulating the muscle decrease related to age [58], and reducing the risk of diabetes type II [4,59]. Chahla et al. [60] reported in their study that osteocalcin is higher in patients who perform regular PA, with an increase in bone mineralization, muscle function, and reduction of risk of diabetes type II.

Moreover, several studies [61,62,63] report that PA could also reduce the level of osteoporosis, resulting in a valid therapeutical approach for this disease in elderly people.

### Limitations

The results of this study should be considered with caution, as there was a high heterogeneity in terms of follow-up, type of intervention, and standardization of physical protocols. In fact, the follow-up varies from a minimum of 1 month to a maximum of 48 months, as well as the number of patients (49 to 392). The small sample size and the high heterogeneity among trials as well as the absence of a control group in three studies [38,41,42], make the estimate of the effect of intervention extremely challenging. Moreover, the low quality of the studies (from “low” to “moderate”), and the high risk of bias of some studies included, decrease the power of our conclusions. Nevertheless, some studies reported an improvement of outcomes in patients treated by PA, even if their results were not statistically significant. These data could lead the authors to overestimate the results considered. Another important limitation of this systematic review is the decision of the authors to include only English and Italian articles. This limitation could lead to an exclusion of relevant studies related to this specific topic. Therefore, further high quality evidences that take into account the standardized methods and a similar cohort of patients are desirable. At the same time, this review should promote future investigations, also including other languages, to better understand which type of PA is preferred to treat older patients with CLBP and help our clinical practice.

## 5. Conclusions

In the available literature PA seems to have a trend of improvement in pain and disability in elderly patients with non-specific CLBP. However, because of the limited and low-quality literature it is not possible to state this positive effect as a definitive conclusion. In order to avoid the overestimated effectiveness of PA on CLBP from high risk of bias studies, new high-quality evidence is needed.

## Figures and Tables

**Table 1:** Cochrane tool for assessing risk of bias in randomized trials (RoB 2 tool).

Unique ID	Randomization process	Deviations From Intended Interventions	Missing Outcome Data	Measurement of the Outcome	Selection of the Reported Result	Overall
Vincent et al. 2014	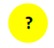	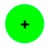	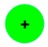	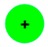	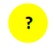	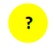
Vincent et al. 2014 II study	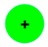	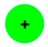	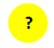	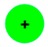	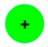	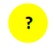
Tsatsako et al. 2016	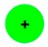	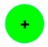	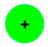	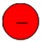	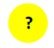	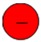
Costantino et al. 2014	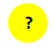	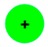	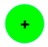	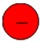	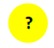	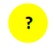
Ferrel et al. 1996	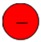	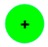	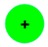	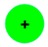	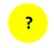	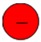
Teut et al. 2016	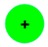	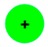	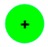	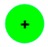	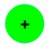	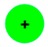
Holmes et al. 1996	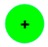	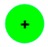	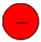	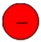	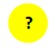	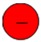

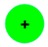
: low risk; 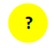: some concern; 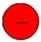: high risk.

**Table 2:** Risk of bias in non-randomized studies of interventions (ROBINS-I).

Unique ID	D1	D2	D3	D4	D5	D6	D7	Overall
Iversen et al.	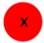	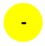	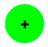	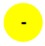	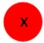	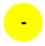	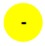	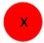
Beissner et al.	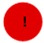	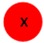	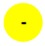	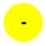	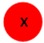	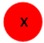	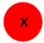	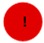
Khalil et al.	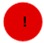	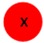	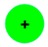	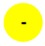	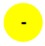	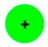	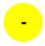	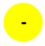
Mailloux et al.	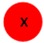	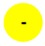	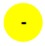	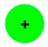	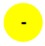	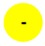	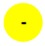	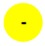
Hicks et al.	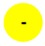	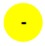	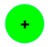	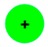	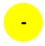	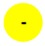	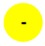	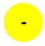

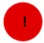
: Critical; 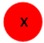: Serious; 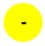: Moderate; 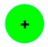: Low.

**Table 3:** GRADE evidence profile.

Certainty Assessment							№ of Patients		Effect	Certainty	Comments
№ of Studies	Study Design	Risk of Bias	Inconsistency	Indirectness	Imprecision	Other Considerations	Physical Activity	NO Intervention	Absolute (95% C.I.)		
Disability RCTs (assessed with: ODI; Scale from: 0% to 100%)											
2 [33,35]	randomized trials	not serious	serious	not serious	serious	none	52	46	MD 1.24% lower (1.94 lower to 0.54 lower), (*p* = 0.0005 *)	⊕⊕◯◯ LOW	PA group shows a lower ODI mean value after treatment. It represents a possible positive influence of PA in improving disability
Disability RCTs (assessed with: SF-36; Scale from: 0 to 100)											
2 [31,32]	randomised trials	not serious	serious	not serious	serious	none	77	67	MD 2.88 point higher (−3.30 lower to 9.6 higher), (p = 0.36)	⊕⊕◯◯ LOW	PA group shows a higher SF-36 mean value after treatment. It represents a possible positive influence of PA in improving disability
Pain RCT (assessed with: NRS; Scale from: 0 to 10)											
1 [36]	randomized trials	not serious	not serious	not serious	serious	none	35	17	MD 1.73 points lower (3.11 lower to 0.35 lower), (*p* = 0.01 *)	⊕⊕⊕◯ MODERATE	PA group shows a lower mean NRS after treatment. It represents a possible positive influence of PA in improving pain
Pain NRCT (assessed with: Global Rating Change; Scale from: 1 to 10)											
1 [34]	observational studies	serious	not serious	not serious	serious	none	238	154	MD 1 points lower (1.53 lower to 0.47 lower), (*p* < 0.001 *)	⊕⊕◯◯ LOW	PA group shows a lower mean pain value after treatment. It represents a possible positive influence of PA in improving pain

C.I.: confidence interval; MD: mean difference; *: statistically significant; NRCT: non-randomized controlled trials; RCT: randomized controlled trials; PA: physical activity; SF-36: 36-Item Short Form Health Survey; ODI: Oswestry Disability Index; NRS: Numerical pain rating scale.

**Table 4:** GRADE summary of findings table.

Outcomes	Anticipated Absolute Effects * (95% C.I.)	№ of Participants (Studies)	Certainty of the Evidence (GRADE)
	Risk with PA		
Disability RCTs assessed with: ODI Scale from: 0% to 100%	MD 1.24% lower (1.94 lower to 0.54 lower), (*p* = 0.0005 *)	98 (2 RCTs) [33,35]	⊕⊕◯◯ LOW
Disability RCTs assessed with: SF-36 Scale from: 0 to 100	MD 2.88 point higher (−3.30 lower to 9.6 higher), (*p* = 0.36)	144 (2 RCTs) [31,32]	⊕⊕◯◯ LOW
Pain RCT assessed with: NRS Scale from: 0 to 10	MD 1.73 points lower (3.11 lower to 0.35 lower), (*p* = 0.01*)	52 (1 RCT) [36]	⊕⊕⊕◯ MODERATE
Pain NRCT assessed with: Global Rating Change Scale from: 1 to 10	MD 1 points lower (1.53 lower to 0.47 lower), (*p* < 0.001 *)	392 (1 observational study) [34]	⊕⊕◯◯ LOW

MD: mean difference, *: statically significant; C.I.: confidence interval.

**Table 5:** Characteristics of the included studies.

Study	Type of Study (LOE)	*n*. of Patients/Mean Age (y)/	Exclusion	Inclusion	Type of Intervention	Control Group	Frequency	Outcome Summary	Outcome Measure/Difference Between Groups	Conclusions
Mailloux et al. [38] 2006	CS (IV)	126/76/48	Compression fracture within the last 6 months, and lack of cognitive or language skills necessary to complete paper-and-pencil measures.	CLBP	Stretching and endurance	No	6 weeks 2 session/week 2 hour/session	Disability Pain	ODI T0–T1 value Physical activity group: 28 (17) to 16 (13) (*p* = 0.01) Control: 38 (17) to 25 (17) (*p* = 0.01)	The exercise behaviours of older adults with CLBP can increase after an exercise-oriented spine physical therapy.
Beissner et al. [39] 2012	OS (II)	59/75.57/2	Not reported	Patients >60 years old; ability to speak English or Spanish; LBP in the past three month, cognitively intact.	Overall fitness: warmup, stretching, endurance exercises, walking, and a cool down	No	9 weeks 2 session/week	Disability	RMDQ T0–T1 values Physical activity group: 3.12 (0.72) to 7.83 (0.77), (*p* < 0.001)	The race/ethnicity could have a role in the improvement of CLBP with a conservative treatment
Iversen et al. [40] 2003	PPIS (III)	26/72/3	Pain with lumbar flexion; low back surgery in the last year; epidural steroid injection during the last 6 months; currently receiving physical therapy or participating in an exercise training program; other medical problems that limited their function more than LBP	Patients >65 years old; low back, buttock, and/or leg pain exacerbated by passive lumbar extension in standing; and symptoms that last for at least 6 months.	Indoor cycling	No	3 months 3 session/week	Disability	SF-36 T0–T1 Physical activity group: 7.2 (*p* = 0.6)	The bicycle program was safe and effective for improving functional status and well-being.
Costantino et al. [41] 2014	RCT (I)	56/73.46/3	Musculoskeletal disorders, cardiac diseases; fever or infectious disease; previous spinal surgery, trauma; previous physical therapies in the last three months	Patients >65 and <80 years old; Diagnosis of chronic non-specific low back pain; Chronic low back pain recurrence in the last three months.	Back school Program	Yes: Hydrotherapy	3 months 2 session/week 1 hour/session	Disability	RMDQ T0–T1 difference Back school: 3.26 ± 1.02, (*p* < 0.001); Hydrotherapy: 4.96 ± 0.71 (*p* < 0.001) SF-36 (Version 2.0) T0–T1 difference Back school: 13.30 ± 1.44 (*p* < 0.001); Hydrotherapy: 14.19 ± 1.98 (*p* < 0.001)	Back School program and Hydrotherapy could be valid treatment options in the rehabilitation of non-specific CLBP in older people.
Ferrel et al. [32] 1997	RCT (I)	33/73/1.5	Unstable cardiovascular or pulmonary diseases, inflammatory arthritis or nerve root compression; psychiatric disease, or alcohol abuse	Age >65 years CLBP, use of analgesic medication; ability to walk independently and able to understand and read English.	Three groups: Group 1: low intensity walking.Group 2: pain education program. Group 3: usual care	Yes: Education programme (one 90- minute session + weekly telephone reinforcement)	6 weeks 4 session/week 1 hour/session	Pain Disability	SF-36 (Version 1.0) T0–T1 difference Intervention: 58.5 (27.7), (*p* < 0.001) Control: 43 (16.7), (*p* < 0.001) PPQ T0–T1 difference Intervention: 28.9 (18.5), (*p* < 0.001) Control: 57.8 (24,9), (*p* < 0.001)	Patient education and fitness walking can improve overall pain management and related functional limitations
Hicks et al. [34] 2012	OS (II)	392/66.8/12	Unstable angina, hypertension, pulmonary disease, dementia, aphasia, back pain attributable to organic causes, back, presence of 2 or more of the following sign: lower-extremity strength, sensation, or reflexes	LBP > 4 months, capability to rise from a chair and walk, capability to travel to the exercise facility, and limited participation in physical activity at the initiation of the exercise program	Strengthening: abdominal strengthening, thoracolumbar, and scapula retraction in lying or standing position or sitting Stretching: hamstring and calf Endurance: 5–10 minutes walking	No	12 months 2 session/week 1 hour/session-20–30 steps	Pain Adherence to exercise Performance	GRC T0–T1 difference Physical activity group: 4.6 (2.5) Control: 4.9 (2.7) (*p* = 0.246)	Patients were able to safely participate in exercise program and back pain improved 12 months later.
Holmes et al. [42] 1996	RCT (I)	38/68.3/3	Not reported	CLBP	Flexion and extension cycles of isotonic resistance exercises	Yes: No exercises	4 weeks, 2 session/week	Pain	VRS T0–T1 values Physical activity group: from 5.3 to 2.1 (*p* < 0.05) Control: data not reported (*p* > 0.05)	In many patients lumbar exercises and resistance exercises could improve CLBP
Khalil et al. [37] 1992	OS (II)	59/68/1	Not reported	In the active restoration program: Low back pain and a diagnosis of myofascial pain syndrome. In the passive restoration program: weakness of quadriceps and/or tibialis anterior.	Mixed isotonic and isokinetic progressive resistive exercise of muscles	No control group The passive approach was based on the use of functional electric stimulation (FES) as an adjunct treatment to strengthen lower extremity muscles weakened by disuse	4 weeks 1 session/day	Pain	Pain level 1–10 T0–T1 values Physical activity group: 5.5 to 3.3 (*p* < 0.01) Control: data not reported	Physical activity can improve symptoms and functional ability of older people that suffer of low back pain. Moreover, FES could be a helpful device in the rehabilitation of weak muscles
Teut et al. [31] 2016	RCT (I)	176/73/3	Acute neurological symptoms within the last 3 months, severe organic or psychiatric disease, metastatic bone disease	Adults ≥65 years old, chronic low back pain for at least 6 months	Yoga group:Viniyoga methodQuijong group:"Dantian“ and Nei Yang Gong exercises from the Training System Liu Ya Fei	Yes: No intervention group	Yoga group: 3 months 24 classes 45 minutes/class Quijong group: 3 months 12 classes 90 minutes/class	Disability	SF-36 T0–T1 value Yoga: 36.3 ± 8.7 to 59.47 (C.I. 54.73; 64.21) Qijong: 37.5 ± 7.8 to 61.01 (C.I. 55.88; 66.14) Control: 36.5 ± 9.3 to 61.17 (C.I. 56.32; 66.02), (*p* = 0.50) FFBH-R T0–T1 value Yoga: 68.7 ± 15.4 to 66.55 (C.I. 62.89; 70.21) Qijong: 70.4 ± 18.7 to 69.23 (C.I. 65.9)	High satisfaction of patients with the yoga and qigong classes, but participation in a 3- or 6-month period of yoga or qigong program did not improve chronic back pain, back function and quality of life.
Tsatsakos [35] et al. 2014	RCT (I)	80/67.7/1	Back surgery, Cauda equina syndrome, spondylolisthesis, rheumatoid conditions	Patients >60 years old, of both sexes and with pain in the lumbar region for a period over 12 weeks	10.000 steps/day performed on a treadmill and during the common life.	Yes: recommendation relaxation, and ergonomic	1 month 8000 steps/day	Disability	ODI T0–T1 value Physical activity group: 7.56 (3.22) to 8.06 (4.94) Control: 11.77 (5.27) to 10.00 (5.03), *p* = 0.46)	Walking shows that it has no effect in the functional status of the elderly with CLBP.
Vincent et al. [33] 2014	RCT (I)	49/67.5/4	Being wheelchair bound	In men and women, BMI ≥30 kg/m^2^, LBP for ≥6 months	Resistance exercise intervention (TOTRX) Lumbar extension intervention (LEXT)	Yes: No intervention	TOTRX: 4 months, 3 session/week 15 exercise/session LEXT: 4 months, 3 session/week 2 sets of lumbar	Disability	ODI T0–T1 values TOTXR: 29.4 (11.2) to 18.0 (12.6) LEXT: 28.6 (15.2) to 22.6 (14.2) Control: 24.4 (12.1) to 22.9 (12.4), (*p* = 0.015) RMDQ	Resistance exercise show improvement in patients walking endurance. Lumbar extension strength in obese older adults with CLBP
Vincent et al. [36] 2014	RCT (I)	49/68.5/4	Wheelchair bound, ability to participate in resistance exercise, acute back pain back surgery within the previous two years	CLBP> 6 months and abdominal obesity and free of abnormal cardiovascular responses during electrocardiogram (ECG) screening tests	TOTRX LEXT	Yes: Behavioural advices: strengthening exercise and nutritional choices	TOTRX: 4 months, 3 session/week 15 sets/exercise/session LEXT: 4 months, 3 session/week 2 sets of lumbar exercises- 15 reps/exercise/session	Pain	NRS T1 value TOTXR: 4.3 (1.8) to 2.0 (1.7) LEXTR: 5.0 (1.8) to 3.7 (2.6) Control: 5.2 (2.4) to 4.6 (2.4), (*p* < 0.006)	Total body resistance exercise (including lumbar extension exercise) was more effective than lumbar extension exercise alone in reducing self-reported disability scores due to back pain

CLBP: chronic low back pain, LBPL low back pain; CS: case-series; LOE: level of evidence; LEXT: lumbar extension intervention; TOTRX: resistance exercise intervention; ODI; Oswestry disability index OS; observational studies; NRS: numerical pain rating scale (NRS); PPIS: pre-post interventional study; PPQ: patient pain questionnaire; RCT: randomized clinical trial, RMDQ: Roland Morris Disability Questionnaire; VRS: Visual rating scale; SF-36: 36-Item short form health survey; FES: functional electric stimulation; T0: baseline values; T1: last follow up values; C.I.: confidence interval; numbers reported in brackets refer to standard deviations.
